# Effects of Plant Regulators on the Seed Germination and Antioxidant Enzyme Activity of Cotton under Compound Salt Stress

**DOI:** 10.3390/plants12244112

**Published:** 2023-12-08

**Authors:** Zhiduo Dong, Jian Huang, Tong Qi, Qiuping Fu, Ajing Meng, Yanbo Fu

**Affiliations:** 1 Institute of Soil Fertilizer, Agricultural Water Saving, Xinjiang Academy of Agricultural Sciences, Urumqi 830091, China; dzd1281228561@163.com (Z.D.); huangjian@xaas.ac.cn (J.H.); maj19890917@163.com (A.M.); fuyanbo2010@163.com (Y.F.); 2 College of Water Conservancy and Civil Engineering, Xinjiang Agricultural University, Urumqi 830052, China; caufuqiuping@126.com; 3 Key Laboratory of Saline-Alkali Soil Improvement and Utilization (Saline-Alkali Land in Arid and Semi-Arid Regions), Ministry of Agriculture and Rural Affairs, Urumqi 830091, China; 4 College of Land Science and Technology, China Agricultural University, Beijing 100193, China; 5 National Soil Quality Aksu Observation Experimental Station, Aksu 843000, China

**Keywords:** 24-epibrassinolide, *Gossypium*, natural composite salts, physiological activity, seedling

## Abstract

Salinity stress significantly hampers cotton seed germination and seedling growth. Employing plant growth regulators stands out as an effective strategy to mitigate salt stress. In this study, we assessed the impact of varying concentrations of natural composite salt conditions (0%, 0.6%, and 1.2%) on cotton seed germination, seedling growth, and physiology. Additionally, we explored the effects of compound sodium nitrophenolate (CSN: 2 mg·L^−1^ and 10 mg·L^−1^), 24-epibrassinolide (EBR: 0.02 mg·L^−1^ and 0.1 mg·L^−1^), and gibberellic acid (GA: 60 mg·L^−1^ and 300 mg·L^−1^), against a control (CK: distilled water) group. The results indicate that with an increase in the composite salt concentration, the germination potential (GP) and germination rate (GR) of cotton seeds gradually decrease. Simultaneously, the fresh weight and root vitality of seedlings also correspondingly decrease, while the degree of membrane lipid peroxidation increases. Under high-salt (1.2%) conditions, soaking treatments with CSN and EBR significantly enhance both GP (45–59% and 55–64%) and GR (30–33% and 39–36%) compared to the CK. However, the GA treatment does not increase the GP and GR of cotton. Moreover, under high-salt (1.2%) conditions, CSN and EBR treatments result in the increased activities of superoxide dismutase (56–66% and 71–80%), peroxidase (20–24% and 37–51%), and catalase (26–32% and 35–46%). Consequently, cotton exhibits a relatively good tolerance to natural composite salts. Soaking treatments with CSN and EBR effectively improve cotton germination by enhancing antioxidant enzyme activities, thereby strengthening cotton’s tolerance to salt stress. These findings offer new insights for enhancing the salt tolerance of cotton.

## 1. Introduction

Soil salinization poses a significant constraint to global agricultural productivity. In China, this challenge extends across approximately 99.13 million hectares, with Xinjiang alone accounting for an astonishing 21.81 million hectares of salinized land [1]. Xinjiang is the main area of cotton planting and production in China, accounting for 83.2% of the total cotton planting area and 90.2% of the country’s overall cotton production [2]. However, the specter of soil salinization hampers the advancement of the cotton industry. When cotton plants face salt stress, particularly during their germination stage, they con-front a heightened vulnerability [3]. The soluble salts present in saline–alkali soil often lead to a decrease in the soil’s osmotic potential, creating a formidable challenge for the absorption of water by plant seeds and roots. This disruption reverberates through the delicate balance of ions within plant cells [4], disrupting internal metabolic processes and substantially sapping the vitality of plant roots and the plant’s photosynthetic capacity [5]. These combined effects directly translate into diminished crop yields. Therefore, the need to enhance salt tolerance during germination becomes paramount for the successful cultivation of cotton in saline–alkali lands.

Soaking seeds in appropriate concentrations of plant growth regulators enhances seed vigor, promotes germination, and expedites seedling growth, leading to improved crop yields, especially under non-biological stress conditions [6]. Currently recognized exogenous plant growth regulators, including gibberellins (hereinafter referred to as GA), compound sodium nitrophenolate (hereinafter referred to as CSN), and 24-epibrassinolide (hereinafter referred to as EBR), have growth-promoting effects. The exogenous application of GA accelerates metabolism and enhances the germination capacity of wheat, marigold, fennel, and cotton seeds under non-biological stress [7,8,9]. CSN enhances protoplasmic streaming, breaks seed dormancy, promotes plant growth and development, and enhances plants’ resistance to salt, low temperature, drought, and heavy metals, ultimately improving crop quality and yield [10,11,12,13]. Under salt stress, maize and *Phaseolus vulgaris* L. seedlings treated with EBR soaking activate antioxidant enzymes by promoting the removal of reactive oxygen species (hereinafter referred to as ROS), contributing to the better maintenance of cell membrane stability and overall plant growth and development [14,15]. These positive effects provide an effective approach to enhancing salt tolerance in cotton.

In the field of cotton salt-tolerance research, studies primarily focus on sodium chlo-ride or artificially created complex salt stress conditions during the germination and seed-ling stages [16,17]. However, the cotton inhabiting natural saline environments often faces a more intricate array of challenges. These conditions significantly differ from artificially simulated salt stress, which usually consists solely of salt components and has a relatively straightforward composition. It is essential to highlight that natural environments feature more diverse soil salt compositions, with various ions such as Na^+^, Ca^2+^, and Mg^2+^ inter-acting among themselves. These interactions may potentially mitigate their impact on plants, and oxidative enzyme activity could exhibit distinct effects [18]. The selection of soil-extracted salts from natural saline soils for plant salt tolerance experiments is a deliberate choice aimed at achieving a more faithful replication of the salt stress effects under natural conditions. This approach takes into account the intricate interplay between soil and salt components. Data obtained through this method can provide valuable insights for agricultural and soil management decision-making, enhancing the applicability of our research findings.

Therefore, this study utilizes a composite salt solution obtained by extracting salts from typical saline–alkali soils in the primary cotton-growing region of southern Xinjiang. The main objectives of this research encompass three key aspects: (1) the evaluation of the influence of compound salt–alkali stress on cotton seed’s germination potential, germination rate, and seedling rate, as well as their fresh weight, osmotic regulatory substances, and antioxidant enzyme activity, among other relevant indicators; (2) an investigation into how various plant growth regulators impact the aforementioned indicators for cotton seeds and seedlings under compound salt–alkali stress conditions; and (3) the assessment of optimal treatments to enhance cotton’s salt tolerance under varying degrees of compound salt–alkali stress.

## 2. Results

### 2.1. Seed Germination

With the increase in salt concentration, both the germination potential and germination rate of cotton seeds exhibit a decreasing trend (Table 1). Compared to the salt-free control (0% CK), the germination potential of the CK decreased by 25% under low-salt (0.6%) conditions, and by 58% under high-salt (1.2%) conditions. Additionally, compared to the salt-free control (0%), the germination rate of the CK decreased by 19% under low-salt (0.6%) conditions and by 43% under high-salt (1.2%) conditions, respectively. Under high-salt (1.2%) conditions, compared to the CK, the germination potential of CSN1, CSN2, EBR1, and EBR2 treatments increased by 45%, 59%, 64%, and 55%, respectively, and the germination rate increased by 30%, 33%, 39%, and 36%, respectively. However, the germination potential of the GA1 and GA2 treatments decreased by 14% and 27%, respectively, and their germination rates decreased by 12% and 21%, respectively. The CSN and EBR treatments enhanced the germination potential and germination rate of cotton seeds, while the GA treatment significantly inhibited seed germination.

### 2.2. Seedling Rate

**Figure 1:**
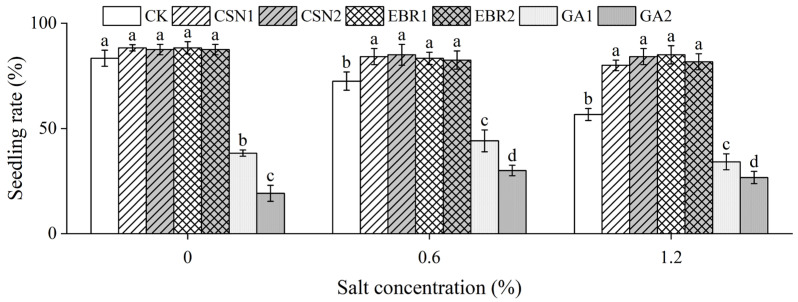
Effects of CSN, EBR, and GA on the seedling rate of cotton under compound salt conditions. Different lowercase letters indicate significant differences at the 0.05 probability level (*p* < 0.05), determined by one-way analysis of variance (ANOVA) and Duncan’s post hoc test for significance. The vertical bar chart represents the mean ± standard deviation (SD) calculated from three repetitions. CK: distilled water soaking treatment; CSN1: soaking treatment with 2 mg·L^−1^ of compound sodium nitrophenolate; CSN2: soaking treatment with 10 mg·L^−1^ of compound sodium nitrophenolate; EBR1: soaking treatment with 0.02 mg·L^−1^ of 24-epibrassinolide; EBR2: soaking treatment with 0.1 mg·L^−1^ of 24-epibrassinolide; GA1: soaking treatment with 60 mg·L^−1^ of gibberellin; GA2: soaking treatment with 300 mg·L^−1^ of gibberellin.

**Figure 2:**
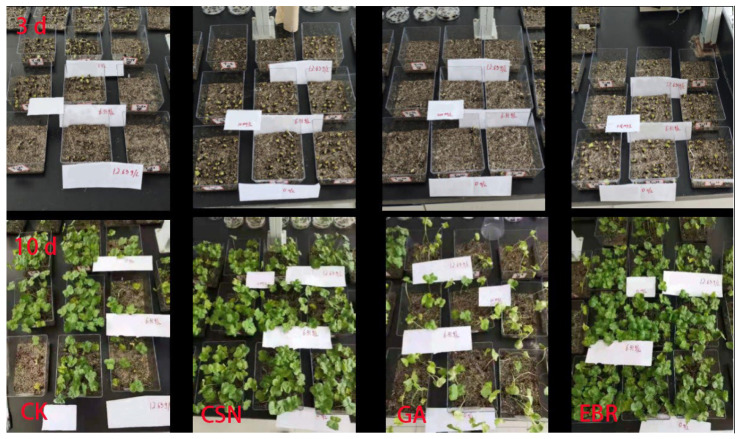
Experimental photographs of the effect of CSN, EBR, and GA on cotton germination and seedlings. where the first row of photographs was taken on the 3rd day after sowing, and the second row was taken on the 10th day after sowing. The first column represents the distilled water control (CK) group, the second column represents the compound sodium nitrophenolate (CSN) treatment group, the third column represents the gibberellic acid (GA) treatment group, and the fourth column represents the 24-epibrassinolide (EBR) treatment group.

The seedling rate of the cotton seeds follows the same trend as the germination potential and germination rate (Figure 1). Compared to the salt-free control (0% CK), the seedling rate of the CK decreased by 13% under low-salt (0.6%) conditions and by 32% under high-salt (1.2%) conditions, respectively. Under high-salt (1.2%) conditions, compared to the CK, the seedling rate of the CSN1, CSN2, EBR1, and EBR2 treatments increased by 41%, 49%, 50%, and 44%, respectively. Figure 2 clearly shows the actual situation of cotton growth. However, the seedling rate of the GA1 and GA2 treatments decreased by 40% and 53%, respectively. The CSN and EBR treatments enhanced the seedling rate of cotton seeds, while the GA treatment significantly inhibited the seedling rate.

### 2.3. Fresh Weight

**Figure 3:**
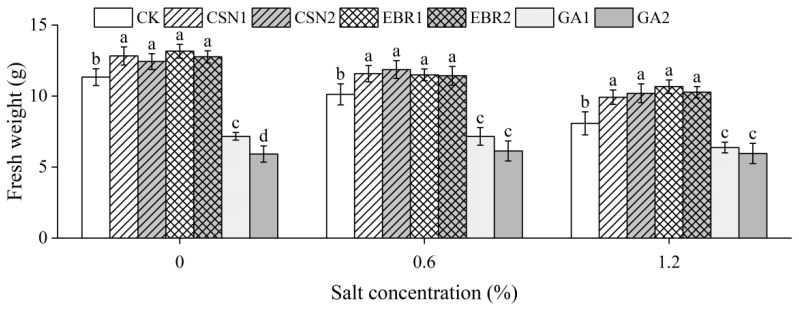
Effects of different treatments on the average fresh weight of cotton seedlings under compound salt conditions. The fresh weight is the average fresh weight of ten cotton seedlings after calculating the average value three times. The error bars represent the standard error (SE) of the mean of three repetitions. Different lowercase letters indicate significant differences at the 0.05 probability level (*p* < 0.05), determined by one-way analysis of variance (ANOVA) and Duncan’s post hoc test for significance. The vertical bar chart represents the mean ± standard deviation (SD) calculated from three repetitions. CK: distilled water soaking treatment; CSN1: soaking treatment with 2 mg·L^−1^ of compound sodium nitrophenolate; CSN2: soaking treatment with 10 mg·L^−1^ of compound sodium nitrophenolate; EBR1: soaking treatment with 0.02 mg·L^−1^ of 24-epibrassinolide; EBR2: soaking treatment with 0.1 mg·L^−1^ of 24-epibrassinolide; GA1: soaking treatment with 60 mg·L^−1^ of gibberellin; GA2: soaking treatment with 300 mg·L^−1^ of gibberellin.

As the salt concentration increases, the fresh weight of the cotton seedlings shows a decreasing trend (Figure 3). Compared to the salt-free control (0% CK), under low-salt (0.6%) and high-salt (1.2%) conditions, the CK’s fresh weight decreased by 11% and 29%, respectively. Under high-salt conditions, both the CSN and EBR treatments significantly increased their fresh weight compared to the CK, with an increase ranging from 23% to 32%. Among them, the EBR1 treatment exhibited the greatest increase in seedling fresh weight. Additionally, both the CSN and EBR treatments also significantly enhanced their seedling fresh weight under salt-free and low-salt conditions.

### 2.4. Dehydrogenase Activity 

**Figure 4:**
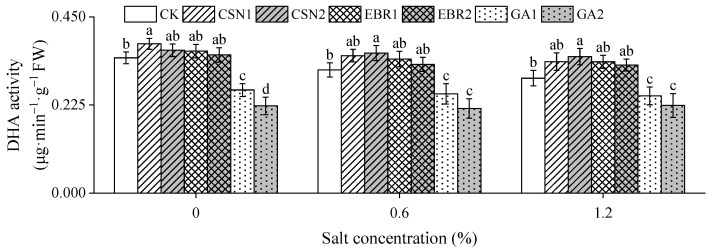
Effects of CSN, EBR, and GA on the dehydrogenase (DHA) activity of cotton seedlings under composite salt conditions. Different lowercase letters indicate significant differences at the 0.05 probability level (*p* < 0.05), determined by one-way analysis of variance (ANOVA) and Duncan’s post hoc test for significance. The vertical bar chart represents the mean ± standard deviation (SD) calculated from three repetitions. CK: distilled water soaking treatment; CSN1: soaking treatment with 2 mg·L^−1^ of compound sodium nitrophenolate; CSN2: soaking treatment with 10 mg·L^−1^ of compound sodium nitrophenolate; EBR1: soaking treatment with 0.02 mg·L^−1^ of 24-epibrassinolide; EBR2: soaking treatment with 0.1 mg·L^−1^ of 24-epibrassinolide; GA1: soaking treatment with 60 mg·L^−1^ of gibberellin; GA2: soaking treatment with 300 mg·L^−1^ of gibberellin.

With the increase in salt concentration, the dehydrogenase (DHA) activity of the cotton seedlings shows a decreasing trend (Figure 4). Compared to the salt-free control (0% CK), under low-salt (0.6%) and high-salt (1.2%) conditions, the CK’s DHA activity decreased by 9% and 15%, respectively. The CSN and EBR treatments increased the DHA activity of seedlings. Under both low-salt and high-salt conditions, the CSN2 treatment exhibited the highest DHA activity, and there were significant differences compared to the CK.

### 2.5. Malondialdehyde Content

**Figure 5:**
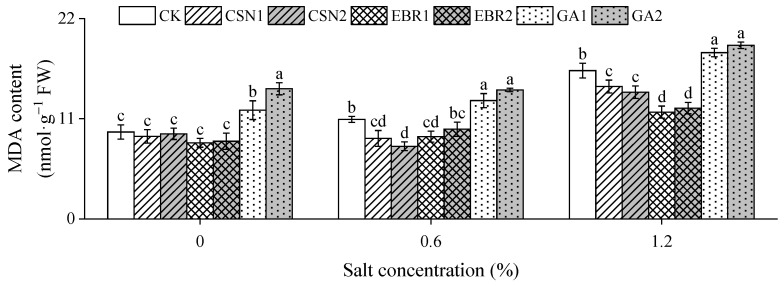
Effects of CSN, EBR, and GA on the malondialdehyde (MDA) content of cotton seedlings under compound salt conditions. Different lowercase letters indicate significant differences at the 0.05 probability level (*p* < 0.05), determined by one-way analysis of variance (ANOVA) and Duncan’s post hoc test for significance. The vertical bar chart represents the mean ± standard deviation (SD) calculated from three repetitions. CK: distilled water soaking treatment; CSN1: soaking treatment with 2 mg·L^−1^ of compound sodium nitrophenolate; CSN2: soaking treatment with 10 mg·L^−1^ of compound sodium nitrophenolate; EBR1: soaking treatment with 0.02 mg·L^−1^ of 24-epibrassinolide; EBR2: soaking treatment with 0.1 mg·L^−1^ of 24-epibrassinolide; GA1: soaking treatment with 60 mg·L^−1^ of gibberellin; GA2: soaking treatment with 300 mg·L^−1^ of gibberellin.

With the increase in salt concentration, the malondialdehyde (MDA) content of the cotton seedlings shows an increasing trend (Figure 5). Compared to the salt-free control (0% CK), under low-salt (0.6%) and high-salt (1.2%) conditions, the CK’s MDA content increased by 14% and 71%, respectively. The CSN and EBR treatments reduced the MDA content of seedlings, particularly under high-salt conditions. Of the high-salt conditions, the EBR1 treatment exhibited the greatest reduction, followed by EBR2, with decreases of 28% and 25% compared to the CK.

### 2.6. Antioxidant Enzyme Activity

**Figure 6:**
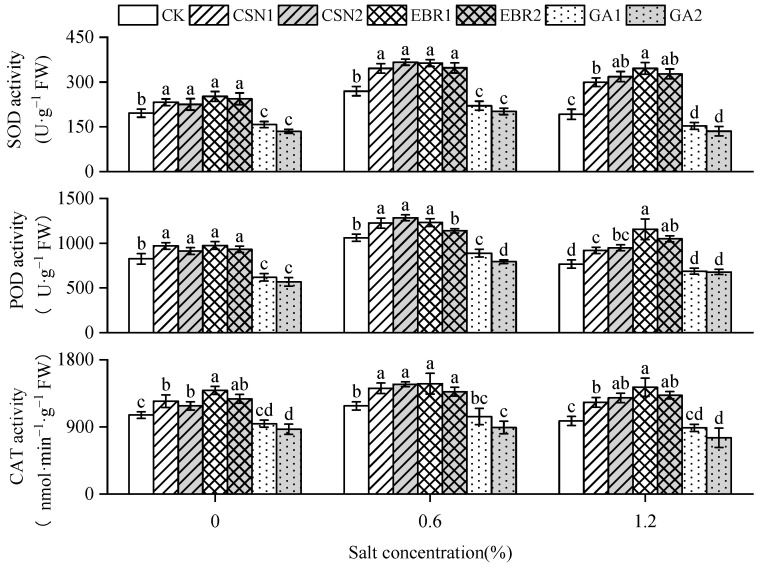
Effects of CSN, EBR, and GA on the superoxide dismutase (SOD), catalase (CAT) and peroxidase (POD) activity of cotton seedlings under composite salt conditions. Different lowercase letters indicate significant differences at the 0.05 probability level (*p* < 0.05), determined by one-way analysis of variance (ANOVA) and Duncan’s post hoc test for significance. The vertical bar chart represents the mean ± standard deviation (SD) calculated from three repetitions. CK: distilled water soaking treatment; CSN1: soaking treatment with 2 mg·L^−1^ of compound sodium nitrophenolate; CSN2: soaking treatment with 10 mg·L^−1^ of compound sodium nitrophenolate; EBR1: soaking treatment with 0.02 mg·L^−1^ of 24-epibrassinolide; EBR2: soaking treatment with 0.1 mg·L^−1^ of 24-epibrassinolide; GA1: soaking treatment with 60 mg·L^−1^ of gibberellin; GA2: soaking treatment with 300 mg·L^−1^ of gibberellin.

With the increase in salt concentration, the activities of superoxide dismutase (SOD), peroxidase (POD), and catalase (CAT) in cotton seedlings exhibit an initial increase followed by a decrease (Figure 6). The CSN and EBR treatments enhance the activities of SOD, POD, and CAT in seedlings, and show significant differences compared to the CK under high-salt conditions. Under high-salt conditions, compared to the CK, the SOD activity of the CSN and EBR treatments increased by 56% to 66% and 71% to 80%, respectively; the POD activity increased by 20% to 24% and 37% to 51%; the CAT activity increased by 26% to 32% and 35% to 46%. Notably, the EBR1 treatment exhibited the highest activities of SOD, POD, and CAT.

### 2.7. Proline Content

**Figure 7:**
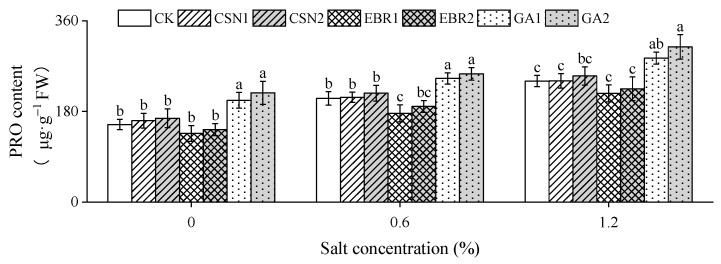
Effects of CSN, EBR, and GA on the proline (PRO) content of cotton seedlings under composite salt conditions. Different lowercase letters indicate significant differences at the 0.05 probability level (*p* < 0.05), determined by one-way analysis of variance (ANOVA) and Duncan’s post hoc test for significance. The vertical bar chart represents the mean ± standard deviation (SD) calculated from three repetitions. CK: distilled water soaking treatment; CSN1: soaking treatment with 2 mg·L^−1^ of compound sodium nitrophenolate; CSN2: soaking treatment with 10 mg·L^−1^ of compound sodium nitrophenolate; EBR1: soaking treatment with 0.02 mg·L^−1^ of 24-epibrassinolide; EBR2: soaking treatment with 0.1 mg·L^−1^ of 24-epibrassinolide; GA1: soaking treatment with 60 mg·L^−1^ of gibberellin; GA2: soaking treatment with 300 mg·L^−1^ of gibberellin.

With the increase in salt concentration, the proline (PRO) content of the cotton seedlings shows an increasing trend (Figure 7). Compared to the salt-free control (0% CK), under low-salt (0.6%) and high-salt (1.2%) conditions, the CK’s PRO content increased by 34% and 56%, respectively. Under high-salt conditions, the PRO content of the CSN and EBR treatments did not significantly differ from the CK, while the PRO content of the GA treatment significantly increased compared to the CK, with GA2 showing the highest increase at 28.24%.

### 2.8. Correlation Analysis

**Figure 8:**
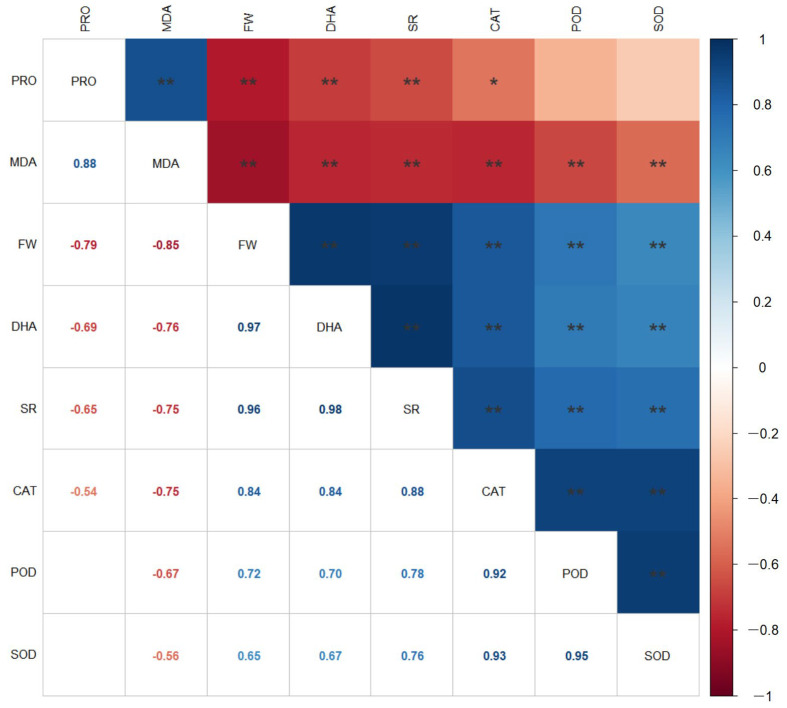
Correlation analysis of the seedling growth rate, seedling biomass, and physiological indexes of cotton seeds. * and ** indicate significant differences at *p <* 0.05 and *p <* 0.01 levels. PRO: proline; MDA: malondialdehyde; FW: fresh weight; DHA: dehydrogenase; SR: seedling rate; CAT: catalase; POD: peroxidase; SOD: superoxide dismutase.

To clarify the correlations between the cotton seedling rate, seedling biomass, and physiological indices, a Pearson correlation analysis was performed on the seedling rate (SR), fresh weight (FW), DHA, MDA, SOD, POD, CAT, and PRO (Figure 8). The analysis revealed that the SR had highly significant positive correlations (*p* < 0.01) with the FW, DHA, SOD, POD, and CAT, while showing highly significant negative correlations with MDA and PRO. This indicates that as the cotton SR increases, there is a simultaneous increase in seedling biomass, antioxidant enzyme activity, and DHA, while the MDA and PRO contents decrease. This reflects the consistency between the cotton seed’s germination and the morphology and functionality of the seedlings.

### 2.9. Comprehensive Evaluation

To avoid the one-sidedness of evaluating based on a single index, a comprehensive assessment, using membership function analysis, was conducted to compare and analyze the mitigating effects of plant growth regulators on the plants’ salt stress relief. Based on the nine indicators of cotton (GP, GR, SR, FW, DHA, MDA, SOD, POD, and CAT) a comprehensive evaluation was performed for each treatment under different salt concentrations. A higher comprehensive evaluation (D) value indicates the better salt tolerance of cotton. From Table 2 it can be observed that, under salt-free conditions, EBR1 ranks the highest, followed by CSN1. Under low-salt conditions, CSN2 ranks the highest, followed by EBR1. Under high-salt conditions, EBR1 ranks the highest, followed by EBR2.

## 3. Discussion

Salinization is a global issue that has adverse effects on crop production. Enhancing crop germination and salt tolerance has become a top priority for addressing this problem. Many researchers are employing various strategies to bolster plants’ resilience to environmental stressors, thus, they are devising agricultural approaches to mitigate these unfavorable conditions [19,20,21]. In order to study the mechanisms that influence crop germination in natural saline–alkali soils, this study utilized a complex salt solution extracted from local saline–alkali soil in Yopurga County. The research findings demonstrated that as the salt concentration increased, the germination rate of the cotton seeds gradually decreased. Complex salt stress significantly inhibited cotton seed germination. Under conditions of high concentrations (1.2%) of complex salt, cotton seed germination rates decreased by 43% compared to the control. In comparison, previous studies by Tao et al. [22] and Li et al. [23] reported a 50% reduction in cotton seed germination rates under single-salt stress with 120 mM (equivalent to 0.70%) and 150 mM (equivalent to 0.87%) NaCl, respectively. Despite similar relative reductions in germination rates (43% and 50%), the natural complex salt concentration was higher than that of NaCl single salt. This suggests that cotton exhibits greater resistance to complex salt stress. Some studies have also indicated that the impact of salt in soil leachate on cotton seed germination is relatively minor [18]. Several factors may contribute to this variation. Firstly, the content of toxic ions (such as Na^+^ and Cl^-^) in the complex salts is lower than that of single salts (NaCl) under the same salt concentration.. For example, in this study, the NaCl content in the high concentration (1.2%) of complex salt was approximately 0.37%, which was lower than the total concentration, potentially reducing ion toxicity. Secondly, antagonistic interactions between different ions within complex salts, such as Ca^2+^, Na^+^, and Mg^2+^ [24,25], may mitigate the toxic effects of single salts. Thirdly, certain ions in complex salts, such as Ca^2+^, are involved in the induction process of salt stress [26], which may enhance the plant’s ability to adapt to salt stress.

Previous studies have shown that EBR seed soaking treatments can enhance cotton seed germination under normal conditions [27], as well as under drought and NaCl stress [28]. While there are relatively fewer studies on the CSN treatment improving cotton seed germination, prior research has indicated that the CSN seed soaking treatment can enhance seed germination in other crops under normal or adverse conditions, such as pepper [29], cucumber [11], and sunflower [30]. Our results demonstrate that both the CSN and EBR treatments increased the cotton seed germination rates, whether under saline or non-saline conditions, with a more significant effect observed, especially under high-salt conditions. This further confirms the substantial positive role of plant growth regulators under more adverse environmental conditions [12]. The CSN and EBR treatments may promote cotton seed germination and seedling establishment by increasing the accumulation of fatty acids within the seeds and enhancing the gene expression associated with fatty acid synthesis, thus bolstering enzyme activity [30,31]. In contrast, in this study, different concentrations of GA, whether under saline or non-saline conditions, exhibited varying degrees of inhibition on cotton seed germination and subsequent growth. This differs from previous research with other plants [32,33,34,35], which showed a growth-promoting effect of GA. Additionally, some studies have indicated that the GA treatment can have a concentration-dependent effect on seed germination and seedling growth, with low concentrations promoting and high concentrations inhibiting these processes. High concentrations of GA can induce osmotic stress, interfere with normal protein and nucleic acid metabolism, and inhibit cell differentiation and development, thereby slowing down plant growth [9]. This might be due to the relatively high concentrations of GA (60–300 mg·L^−1^) used in this study, which resulted in the suppression of cotton seed germination.

When plants are subjected to salt stress, they employ an osmotic tolerance strategy [36] to mitigate the impact of high salt concentrations on their water uptake. This strategy involves absorbing a large amount of salt ions to increase their cells’ osmotic potential, thus enhancing their water absorption capacity [20,37]. During this process, excessive salt accumulation exerts toxic effects on the internal structure of plant seeds, potentially causing changes in protein synthesis, energy production, and respiration [21]. This can also lead to alterations in hormone and nutrient balance during seed germination, thus affecting the normal germination process of seeds [6,21,23]. High concentrations of salt ions in cells adversely affect multiple biological processes [5], including disrupting transmembrane electrochemical gradients, leading to the increased generation of reactive oxygen species (ROS) [38,39,40]. Excess ROS can lead to lipid membrane oxidation, affecting normal cellular physiological functions. Malondialdehyde (MDA) is the end product of membrane lipid peroxidation and serves as a crucial indicator reflecting the extent of the cell membrane damage caused by ROS [14,41,42]. Our study results indicate that as the concentration of complex salts increases, the MDA content in cotton leaves also increases. This suggests that cotton experiences membrane lipid oxidation and damage under complex salt stress, which aligns with the findings in a study conducted under NaCl stress [23]. However, at the same concentration, both the CSN and EBR seed soaking treatments reduced the MDA content in cotton seeds compared to the control. Some studies suggest that exogenous EBR application helps inhibit the generation of reactive oxygen species and reduce electrolyte leakage, thereby improving membrane stability [42]. EBR seed soaking treatments have effectively mitigated oxidative damage in maize and mustard seedlings under salt stress, resulting in their reduced MDA content [14,43]. Previous reports indicate that CSN seed soaking treatments effectively alleviate the oxidative damage in cucumber seedlings under cold stress, leading to their decreased MDA content [11]. Therefore, it can be inferred that the application of CSN and EBR is beneficial in reducing membrane damage under salt stress. This may be due to the induction of oxidative stress responses by CSN and EBR in plants, which, to some extent, counteracts the oxidative effects of ROS. Plants possess antioxidant defense systems that effectively eliminate ROS, primarily consisting of enzymes such as SOD, POD, and CAT. SOD plays a significant role as a scavenger of reactive oxygen in plant cells, converting superoxide into H_2_O_2_ and molecular oxygen [41,44]. Subsequently, CAT and POD convert H_2_O_2_ into water and oxygen. These enzymes work together to clear the MDA generated by lipid peroxidation to protect the membrane structure [44]. However, as the salt concentration increases, there were differences in the trend of antioxidant enzyme (SOD, POD, and CAT) activity in cotton, which was related to cotton varieties, salt concentration and stress time [45,46]. In this study, under saline conditions, both the CSN and EBR treatments enhanced the antioxidant enzyme activity of cotton seedlings, particularly under high-salt (1.2%) stress, where the SOD, POD, and CAT activity in cotton seedlings treated with 0.02 mg·L^−1^ EBR was higher than in other treatments. This aligns with the results of Rattan et al. [14], who applied EBR to maize seeds and subsequently observed an increase in SOD, POD, and CAT activity in maize seedlings under NaCl stress. Moreover, EBR has been reported to activate various antioxidant enzyme activities in several plants, including bean, tomato, and maize [14,15,42,47,48]. Previous research has shown that EBR can regulate antioxidant gene expression and act as a signaling compound to activate both enzymatic and non-enzymatic antioxidants [42]. This further indicates that the EBR treatment can mitigate oxidative damage directly or indirectly.

## 4. Materials and Methods

### 4.1. Experimental Location and Materials

The experiment was conducted at the Soil Fertilizer and Agricultural Water Conservation Laboratory of Xinjiang Academy of Agricultural Sciences from June to August 2021. The cotton cultivar used was XinLuZhong 49 (L, Gossypium hirsutum L.), provided by the Institute of Economic Crops at Xinjiang Academy of Agricultural Sciences. Compound sodium nitrophenolate (CSN) was sourced from Zhengzhou Nong-da Biochemical Products Co., Ltd. (Zhengzhou, China). Gibberellin (GA) was obtained from Sichuan Lomon Bio Technology Co., Ltd. (Meishan, China). 24-epibrassinolide (EBR) was acquired from Hebei Lan sheng Biotechnology Co., Ltd. (Shijiazhuang, China). The composite salt solution was prepared by diluting a 7.5% high-salt solution obtained through leaching salt–alkali soil from Yuepuhu County (Longitude: 76.76926°, Latitude: 39.19173°), Kaxgar, with a salt content of 80 g·kg^−1^. The pH value, mineralization, and concentrations of the eight major ions are presented in Table 3. The pH value was determined using a digital pH meter (pHS.25 type), and conductivity was measured using a conductivity meter (PE38). The ion determination methods used were as follows: HCO_3_^−^ and CO_3_^2−^ were determined using double indicator titration, Cl^−^ was determined through AgNO_3_^−^ titration, SO_4_^2−^ was determined through EDTA indirect titration, Ca^2+^ and Mg^2+^ were determined through EDTA complexometric titration, and K^+^ and Na^+^ were determined using flame photometry.

### 4.2. Cotton Seed Treatment

Plump and uniform cotton seeds were selected for the experiment. The seeds were first immersed in a 95% anhydrous ethanol solution for 1 min to ensure disinfection. They were then rinsed with distilled water five times and gently dried using filter paper to remove excess moisture from the seed surface. Subsequently, the treated seeds were divided into different groups based on the experimental design. Each group was soaked in specific solutions for 20 h as follows: CSN1, 2 mg·L^−1^ of compound sodium nitrophenolate; CSN2, 10 mg·L^−1^ of compound sodium nitrophenolate; EBR1, 0.02 mg·L^−1^ of 24-epibrassinolide; EBR2, 0.1 mg·L^−1^ of 24-epibrassinolide; GA1, 60 mg·L^−1^ of gibberellin; and GA2, 300 mg·L^−1^ of gibberellin. Distilled water was used as a control and the seeds were soaked in it for 20 h. After the soaking process, the cotton seeds were placed on an ultra-clean table for drying at a temperature of 25 °C, indoors, for 24 h.

### 4.3. Experimental Design

The salt concentrations were set at 0.6% and 1.2%, based on the ‘Soil Agrochemical Analysis’ [49], with distilled water used as a control. The experiment consisted of two parts: a seed germination test and seedling growth test. Both tests followed a consistent approach, involving two different concentrations of plant growth regulators soaked under three composite salt conditions (0%, 0.6%, and 1.2% salt concentrations). Distilled water was used as the control.

#### 4.3.1. Seed Germination Test

After soaking, plump and uniform seeds were placed in Petri dishes (diameter: 10 cm) lined with three layers of filter paper (80 ± 4 g/m^2^) (Hangzhou SPECIAL Paper Industry Co., Ltd. Suzhou, China). Each dish contained 20 seeds and was treated with 10 mL of composite salt solution at varying salt concentrations (0%, 0.6%, and 1.2%). The indoor temperature was maintained at 25 °C to facilitate germination. Each treatment was replicated three times, resulting in a total of 21 treatments.

#### 4.3.2. Seedling Growth Test

After soaking, the seeds were sown in sterilized vermiculite (moisture content of 32.5%) culture boxes, with 40 seeds in each box. The vermiculite layer had a uniform thickness of 1 cm and was evenly perforated with 5 × 8 small holes. These holes had a depth of 0.8 cm and were spaced equally to prevent seed contact. Each box was irrigated with 100 mL of composite salt solution at different salt concentrations (0%, 0.6%, and 1.2%). The temperature was maintained at 25 °C for indoor cultivation. Each treatment was replicated three times, resulting in a total of 21 treatments.

### 4.4. Estimation of Plant Growth Parameters 

A sprout length exceeding half of the seed length was considered germination [50], while a seedling height of more than 3 cm was considered successful establishment [51]. The germination potential (GP), germination rate (GR), and seedling rate (SR) were calculated according to the following formulas:

(1) $$GP\left(\%\right)=\left(\frac{N_{3}}{N}\right)\times 100\%$$

(2) $$GR\left(\%\right)=\left(\frac{N_{7}}{N}\right)\times 100\%$$

(3) $$SR\left(\%\right)=\left(\frac{M_{14}}{M}\right)\times 100\%$$

where *N*_3_ is the number of germinated cotton seeds in the culture box on the 3rd day after sowing, *N*_7_ is the number of germinated cotton seeds in the culture box on the 7th day after sowing, *N* is the total number of cotton seeds sown in the culture box, *M*_14_ is the number of cotton seedlings in the culture box on the 14th day after sowing, and *M* is the total number of cotton seeds sown in the culture box.

On the 14th day of the seedling growth test, 10 seedlings were randomly selected from each treatment and weighed using a balance with a precision of 0.01%. If there were fewer than 10 seedlings available, the utmost effort was made to select as many seedlings with consistent growth as possible. Each treatment was replicated three times.

### 4.5. Estimation of Root Viability

To determine the root vitality of the cotton seedlings, the procedure was carried out following the method of Su et al. [52]. Initially, 0.2 g of roots were placed in a 10 mL solution containing 0.06 mM sodium phosphate (Na_2_HPO_4_-KH_2_PO_4_) with 0.4% (*w*/*v*) TTC (2,3,5-triphenyltetrazolium chloride). Vacuum infiltration was conducted for 15 min at 37 °C, followed by an incubation of 10 h. Subsequently, the samples were extracted using 95% (*v*/*v*) ethanol and then incubated at 90 °C in a water bath for 15 min. Absorbance was recorded at 520 nm to determine dehydrogenase (DHA) activity.

### 4.6. Estimation of Lipid Peroxidation

To determine the degree of lipid peroxidation in the cotton leaves, we first took 0.5 g of leaves and incubated them with 5 mL of thiobarbituric acid (TBA) at 95 °C in a hot water bath. After 30 min of incubation, the mixture was transferred to a new transparent test tube and placed in a cooler with ice for proper cooling. Subsequently, it was centrifuged at 10,000× *g* and 25 °C for 10 min. Next, 200 μL of the supernatant was drawn and placed in a microquartz cuvette or a 96-well plate, and absorbance at 532 nm and 600 nm was measured using a UV-visible spectrophotometer. Finally, the content of malondialdehyde (MDA) was estimated by calculating the difference in absorbance between 532 nm and 600 nm, in order to determine the degree of lipid peroxidation in the cotton leaves.

### 4.7. Antioxidant Enzyme Activity Determination

To analyze antioxidant enzymes, we used 0.5 g of cotton seedling leaves. Firstly, fresh leaf tissue was ground in a cold water bath and mixed with a 0.05 mol/L phosphate-buffered saline (pH 7.4). Subsequently, it was centrifuged at 8000× *g* for 10 min at 4 °C. The resulting supernatant was used for the subsequent analysis of the antioxidant substance content.

To determine the activity of superoxide dismutase (SOD), peroxidase (POD), and catalase (CAT), we utilized specific assay kits, namely SOD-1-W, POD-1-Y, and CAT-1-Y, respectively. These assay kits were provided by Suzhou Kemeing Biotechnology Co., Ltd. (Suzhou, China).

### 4.8. Estimation of Proline Content

To determine the proline content, we first took 0.5 g of leaves and mixed them with 5 mL of a 3% sulfosalicylic acid solution, ensuring that the process was carried out under cold conditions. The mixing was performed using a mortar and pestle, following the method described by Mahmud et al. [53]. Subsequently, we placed the mixture in a centrifuge and centrifuged it at 10,000× *g* for 12 min to obtain the supernatant. Next, we took 1 mL of the supernatant and mixed it thoroughly with 1 mL of acid ninhydrin and 1 mL of glacial acetic acid. The mixture was then incubated at 95 °C in a hot water bath for 10 min. After the incubation, the mixture solution was transferred to a clean test tube and placed in an ice-containing box for proper cooling. Following that, 2 mL of toluene was added to the cooled solution, and the solution was thoroughly vortexed. Finally, we recorded the absorbance of the toluene containing the chromophore, spectrophotometrically, at a wavelength of 520 nm, and the proline content was estimated using a standard curve generated from known concentrations.

### 4.9. Statistical Analysis

Experimental data management was performed using Excel 2016; one-way analysis of variance (ANOVA) and Duncan’s multiple comparisons were conducted using SPSS 26.0, and the charts were created using Origin 2018. Pearson correlations were calculated for the cotton seedling rate, seedling fresh weight, and physiological indicators, and correlation plots were generated using the ‘cor’ function and ‘corrplot’ package within R (v.4.3.1 for Windows) and RStudio IDE (2 June 2023).

A fuzzy mathematics membership function method was employed to comprehensively evaluate the cotton seed germination, seedling fresh weight, and physiological indicators under different salinity levels. The membership function formula is as follows:

(4) $$U\left(X_{i}\right)=\frac{X_{i}-X_{min}}{X_{max}-X_{min}}$$

where U($X_{i}$) represents the membership function value, $X_{i}$ is the measured value of a specific indicator at a treatment level, and $X_{min}$ and $X_{max}$ are the minimum and maximum values of that indicator within all experimental levels.

If a specific indicator is negatively correlated with stress resistance, a reverse membership function is used for quantitative transformation, and the calculation formula is as follows:

(5) $$U\left(X_{i}\right)=1-\left[\frac{X_{i}-X_{min}}{X_{max}-X_{min}}\right]$$

## 5. Conclusions

With the increasing salinity, the inhibitory effect of natural complex salt on cotton (XinLuZhong 49) germination and seedling growth becomes significantly more pronounced, but this inhibitory effect is less than that of single salts. CSN and EBR seed soaking treatments promote cotton seed germination and enhance seedling salt tolerance by increasing their antioxidant enzyme activity, reducing cell membrane lipid oxidation. In this study, GA did not show an improvement in cotton salt tolerance. We utilized the method of subordinate functions to comprehensively analyze and evaluate the optimal hormone concentrations, in this experiment, that can promote cotton germination.

## Figures and Tables

**Table 1:** Effects of different plant regulator treatments on the germination potential and germination rate of cotton seeds under compound salt conditions.

Salt Concentration (%)	Treatment	Germination Potential (%)	Germination Rate (%)
0	CK	88.33 ± 2.89 b	96.67 ± 2.89 a
	CSN1	95 ± 5 a	98.33 ± 2.89 a
	CSN2	93.33 ± 2.89 ab	98.33 ± 2.89 a
	EBR1	96.67 ± 2.89 a	100 ± 0 a
	EBR2	93.33 ± 2.89 ab	98.33 ± 2.89 a
	GA1	48.33 ± 2.89 c	63.33 ± 2.89 b
	GA2	23.33 ± 2.89 d	31.67 ± 2.89 c
0.6	CK	66.67 ± 2.89 b	78.33 ± 2.89 b
	CSN1	78.33 ± 2.89 a	90 ± 0 a
	CSN2	83.33 ± 2.89 a	91.67 ± 2.89 a
	EBR1	78.33 ± 2.89 a	90 ± 0 a
	EBR2	78.33 ± 2.89 a	88.33 ± 2.89 a
	GA1	53.33 ± 2.89 c	66.67 ± 2.89 c
	GA2	33.33 ± 2.89 d	53.33 ± 2.89 d
1.2	CK	36.67 ± 2.89 c	55 ± 5 b
	CSN1	53.33 ± 2.89 b	71.67 ± 2.89 a
	CSN2	58.33 ± 2.89 ab	73.33 ± 2.89 a
	EBR1	60 ± 5 a	76.67 ± 2.89 a
	EBR2	56.67 ± 2.89 ab	75 ± 5 a
	GA1	31.67 ± 2.89 cd	48.33 ± 2.89 bc
	GA2	26.67 ± 2.89 d	43.33 ± 2.89 c

Note: Different lowercase letters indicate significant differences at the 0.05 probability level (*p* < 0.05), determined by one-way analysis of variance (ANOVA) and Duncan’s post hoc test. The data are presented as means ± standard deviation (SD) calculated from three repetitions. CK: distilled water soaking treatment; CSN1: soaking treatment with 2 mg·L^−1^ of compound sodium nitrophenolate; CSN2: soaking treatment with 10 mg·L^−1^ of compound sodium nitrophenolate; EBR1: soaking treatment with 0.02 mg·L^−1^ of 24-epibrassinolide; EBR2: soaking treatment with 0.1 mg·L^−1^ of 24-epibrassinolide; GA1: soaking treatment with 60 mg·L^−1^ of gibberellin; GA2: soaking treatment with 300 mg·L^−1^ of gibberellin.

**Table 2:** Analysis and comprehensive evaluation of the membership function of each index of cotton.

Salt Concentration	Treatment	Membership Function									D Value	Rank
		GP	GR	SR	FW	DHA	MDA	SOD	POD	CAT		
0	CK	0.89	0.95	0.93	0.75	0.77	0.80	0.52	0.63	0.36	0.73	5
	CSN1	0.98	0.98	1.00	0.95	1.00	0.88	0.83	0.99	0.72	0.93	2
	CSN2	0.95	0.98	0.99	0.90	0.90	0.84	0.77	0.85	0.60	0.86	4
	EBR1	1.00	1.00	1.00	1.00	0.88	1.00	1.00	1.00	1.00	0.99	1
	EBR2	0.95	0.98	0.99	0.95	0.82	0.97	0.93	0.90	0.78	0.92	3
	GA1	0.34	0.46	0.28	0.17	0.26	0.40	0.20	0.13	0.14	0.26	6
	GA2	0.00	0.00	0.00	0.00	0.00	0.00	0.00	0.00	0.00	0.00	7
0.6	CK	0.67	0.65	0.77	0.70	0.70	0.52	0.41	0.54	0.49	0.61	5
	CSN1	0.90	0.96	0.98	0.95	0.95	0.86	0.87	0.88	0.90	0.92	3
	CSN2	1.00	1.00	1.00	1.00	1.00	1.00	1.00	1.00	0.99	1.00	1
	EBR1	0.90	0.96	0.97	0.93	0.91	0.83	0.98	0.89	1.00	0.93	2
	EBR2	0.90	0.91	0.95	0.92	0.81	0.70	0.89	0.70	0.82	0.84	4
	GA1	0.40	0.35	0.26	0.18	0.33	0.19	0.12	0.19	0.25	0.25	6
	GA2	0.00	0.00	0.00	0.00	0.19	0.00	0.00	0.00	0.00	0.02	7
1.2	CK	0.30	0.35	0.51	0.45	0.56	0.38	0.27	0.19	0.33	0.37	5
	CSN1	0.80	0.85	0.91	0.84	0.90	0.62	0.78	0.50	0.70	0.77	4
	CSN2	0.95	0.90	0.99	0.90	1.00	0.70	0.87	0.57	0.79	0.85	3
	EBR1	1.00	1.00	1.00	1.00	0.89	1.00	1.00	1.00	1.00	0.99	1
	EBR2	0.90	0.95	0.94	0.92	0.83	0.94	0.91	0.78	0.84	0.89	2
	GA1	0.15	0.15	0.13	0.09	0.20	0.11	0.08	0.02	0.20	0.12	6
	GA2	0.00	0.00	0.00	0.00	0.00	0.00	0.00	0.00	0.00	0.00	7

Note: GP: germination potential; GR: germination rate; SR: seedling rate; FW: fresh weight; DHA: dehydrogenase; MDA: malondialdehyde; SOD: superoxide dismutase; CAT: catalase; POD: peroxidase; D: comprehensive evaluation. CK: distilled water soaking treatment; CSN1: soaking treatment with 2 mg·L^−1^ of compound sodium nitrophenolate; CSN2: soaking treatment with 10 mg·L^−1^ of compound sodium nitrophenolate; EBR1: soaking treatment with 0.02 mg·L^−1^ of 24-epibrassinolide; EBR2: soaking treatment with 0.1 mg·L^−1^ of 24-epibrassinolide; GA1: soaking treatment with 60 mg·L^−1^ of gibberellin; GA2: soaking treatment with 300 mg·L^−1^ of gibberellin.

**Table 3:** pH value, mineralization degree and eight ion contents of the salt solution.

Title	Unit	Low-Salt (0.6%)	High-Salt (1.2%)
pH	—	7.25	7.19
Salinity	g·L^−1^	6.2	12.4
electrical conductivity	S·m^−1^	1.041	1.785
HCO_3_^−^	g·kg^−1^	0.2348	0.1981
Cl^−^	g·kg^−1^	2.0046	3.7661
SO4^2−^	g·kg^−1^	3.0816	6.3686
Ca^2+^	g·kg^−1^	0.1284	0.3082
Mg^2+^	g·kg^−1^	0.0783	0.0731
K^+^	g·kg^−1^	0.0337	0.0455
Na^+^	g·kg^−1^	2.547	4.125

## Data Availability

Data are contained within the article.
